# Aerobic Responses to FES-Assisted and Volitional Cycling in Children with Cerebral Palsy

**DOI:** 10.3390/s21227590

**Published:** 2021-11-15

**Authors:** Ashwini Sansare, Ann Tokay Harrington, Henry Wright, James Alesi, Ahad Behboodi, Khushboo Verma, Samuel C. K. Lee

**Affiliations:** 1 Biomechanics and Movement Science Program, University of Delaware, Newark, DE 19713, USA; ashwini@udel.edu (A.S.); vkhush@udel.edu (K.V.); 2 Department of Physical Therapy, University of Delaware, Newark, DE 19713, USA; henryw@udel.edu (H.W.); jfalesi@udel.edu (J.A.); 3 Department of Physical Therapy, Arcadia University, Glenside, PA 19038, USA; harringtona@arcadia.edu; 4 Functional and Applied Biomechanics Section, Rehabilitation Medicine Department, National Institutes of Health, Bethesda, MD 20892, USA; ahad.behboodi@nih.gov

**Keywords:** cardiorespiratory fitness, recumbent cycling, tricycle, biking

## Abstract

Recumbent stationary cycling is a potential exercise modality for individuals with cerebral palsy (CP) that lack the postural control needed for upright exercises. Functional electrical stimulation (FES) of lower extremity muscles can help such individuals reach the cycling intensities that are required for aerobic benefits. The aim of this study was to examine the effect of cycling with and without FES assistance to that of a no-intervention control group on the cardiorespiratory fitness of children with CP. Thirty-nine participants were randomized to a FES group that underwent an 8-week FES-assisted cycling program, the volitional group (VOL), who cycled without FES, or a no-intervention control group (CON) (15 FES, 11 VOL, 13 CON). Cadence, peak VO_2_, and net rise in heart rate were assessed at baseline, end of training, and washout (8-weeks after cessation of training). Latent growth curve modeling was used for analysis. The FES group showed significantly higher cycling cadences than the VOL and CON groups at POST and WO. There were no differences in improvements in the peak VO_2_ and peak net HR between groups. FES-assisted cycling may help children with CP attain higher cycling cadences and to retain these gains after training cessation. Higher training intensities may be necessary to obtain improvements in peak VO_2_ and heart rate.

## 1. Introduction

Individuals with cerebral palsy (CP) present with reduced muscle strength, muscle tone abnormalities, co-contraction of agonist and antagonist muscles, and poor selective voluntary motor control [1,2]. These aforementioned impairments result in poor motor performance and reduced mobility in children with CP. As children with CP enter adolescence and adulthood, there is a decline in independence, limited participation in physical activities and sports, and poor cardiorespiratory fitness compared to their peers [3,4,5]. Furthermore, limited accessibility to community fitness resources, recreational activities, exercise equipment, and lack of appropriate physical education programs in schools form additional barriers to engaging in a physically active lifestyle [3,5,6,7]. Thus, there is a critical need to identify and develop safe and effective means of improving and maintaining physical fitness levels in children with CP.

Recumbent cycling is a potential exercise modality for individuals lacking the strength or postural control necessary for exercises in standing or walking positions [8,9]. Abnormal muscle tone and motor activation, however, may prevent individuals with CP from attaining cycling cadences and heart rates required to produce aerobic benefits. Functional electrical stimulation (FES), which involves appropriately timed electrical muscle contractions for activity, may help such individuals achieve that cycling intensities that are necessary for aerobic benefits that are otherwise unachievable on their own. FES-assisted cycling improved fitness, bone mineral density, and muscle mass in individuals with spinal cord injuries [10,11,12,13,14]. In children with CP, several case studies have shown improved cadence, power output, heart rate, and muscle strength and reduced co-contraction and oxygen expenditure following FES training [15,16,17]. In a recent randomized control trial (RCT), Armstrong et al. showed that an FES-based cycling combined with goal-directed training improved gross motor function and self-reported goal performance and satisfaction [18]. However, there are no larger RCTs that have assessed the effect of FES cycling on cardiorespiratory measures. Additionally, it is not known if FES assistance may provide cardiorespiratory benefits over and above a cycling-only protocols since these benefits could be attributed to improved fitness due to cycling and not FES-assistance.

The aim of this study was to examine the effect of two training approaches, cycling with and without FES assistance, to that of a no-intervention control group on the cardiorespiratory fitness of children with CP. Specifically, this study investigated group differences between participants undergoing an FES-assisted cycling training protocol (FES group), participants undergoing volitional cycling only (VOL group), and participants receiving no intervention (CON group) on cadence, peak oxygen uptake (peak VO_2_), and heart rate (HR) measures (1) following 8-weeks of cycling training and (2) after an 8-week washout period to assess the retention of the training effects. The hypothesis was that the FES group would show the greatest increase in cadence, peak VO_2_, and heart rate following the intervention period followed by VOL and lastly by CON. Additionally, it was hypothesized that the FES group would show the greatest retention in their peak VO_2_, heart rate, and cadence gains after a washout period followed by VOL and lastly by CON.

## 2. Materials and Methods

### 2.1. Study Design

A parallel three-group, randomized, cross-sectional experimental design was used. Based on previously collected pilot data, an a priori power analysis determined a sample size of 60 subjects, with approximately 20 subjects per group needed to produce significant results. Appropriate institutional review board permissions were obtained. All study procedures were explained, and all participants signed informed assent or consent documents (if 18 years old), and a parent or legal guardian signed consent documents for minors.

### 2.2. Participants

Children and young people with spastic diplegic CP between the ages of 10–18 years were recruited from the outpatient CP clinic at Shriners Hospitals for Children, Philadelphia, and local referral sources. All individuals were screened by a physical therapist and an orthopedic surgeon for the inclusion and exclusion criteria (Table 1) and were randomized to one of three groups: FES, VOL, or CON. Block randomization was used to ensure equal allocation across the three groups. Sequentially numbered, opaque, sealed envelopes in block sizes of three, with one envelope each for FES, VOL, and CON in random order within each block, were opened after participant consent was obtained. The participants and the research team were not blinded.

### 2.3. Instrumentation

Participants in the FES and VOL groups were trained on commercial recumbent sport tricycles (KMXKart, Birmingham, UK) instrumented with sensors to enable the calculation of the cycling power output [15]. The tricycles were mounted on a stationary trainer, and those receiving FES assistance included a tricycle mounted FES stimulator. The tricycle crank and spindle assembly were instrumented with a torque sensor and encoder to measure the torque applied during each pedal stroke, calculate power output, and to indicate crank position and cadence. During testing assessments, a similarly instrumented tricycle was used. For the FES group, a RehaStim stimulator (Hasomed GmbH, Magdeburg, Germany) was controlled by custom software (The Math Works Inc., MATLAB) to apply stimulation to the bilateral quadriceps femoris muscles. FES was applied at a current of 40 mA and a frequency of 50 Hz via transcutaneous electrodes while the FES software modulated the stimulus pulse duration. The quadriceps muscle was stimulated (via MATLAB Simulink control) in coordination with the crank angle of the tricycle during the “pushing phase”, i.e., the phase during which the hip and knee went from maximum flexion to moderate hip-knee extension (the arc of cycling motion when the pedal was located between ~40 degrees before and ~70 degrees past top dead center, respectively).

### 2.4. Procedure

#### 2.4.1. Set-Up Phase

Because recumbent cycling was a novel activity for the children with CP, all of the participants underwent a set-up/practice phase prior to baseline testing (three 20 min cycling sessions in a laboratory setting). For both of the treatment groups, the participants and their caregivers were trained on system setup and how to implement the cycling training protocol at home. For the FES group, the participants practiced cycling with a gradual increase in the intensity of the stimulation until the maximum tolerable pulse duration was achieved. The pulse durations corresponding to the sensory perception of the stimulation and the maximum tolerable intensity were recorded and entered into the individual’s custom FES cycling program.

An incremental cycling exercise test was performed during the initial visit to determine the power output range that corresponded with the 50–80% Karvonen target heart rate (details of which are under the section on Outcome Measures). This range of power output was later used as a target to guide the cycling intensity during the intervention phase for FES and VOL groups via visual feedback displayed on a laptop. Cadence, peak VO_2_, and peak net rise in heart rate were all obtained during the incremental cycle test. The participants from the FES group were sent home with an FES tricycle system and a Polar heart rate monitor (E600, Polar Electro, Kempele, Finland), while those in the VOL group were sent home with the same systems excluding the stimulator. A home-training log was maintained by each family. For both cycling groups, percentage adherence was calculated by dividing the number of sessions logged during the training period by the total expected number of sessions (24 sessions).

#### 2.4.2. Training Protocol

##### FES Group

The participants were asked to cycle continuously for 30 min three times a week for 8 weeks at the target cycling power corresponding to 50–80% of their Karvonen-predicted target heart rate during the baseline incremental test. Electrodes were placed over the quadriceps muscles of both legs and the stimulation settings for FES determined during the set-up phase were used. Exercise was performed while using a video game graphic on a laptop to help encourage the maintenance of the prescribed power output. The video game consisted of an airplane whose vertical height was controlled by the cycling power generated by the participant; the center of the screen corresponded to the participant’s specific target cycling power. The participant was prompted to cycle and to maintain the airplane in the center of the screen. If the participant was unable to attain the target power, the FES-stimulation ramped from the sensory level (the pulse duration that produced cutaneous sensation) to a motor level that assisted the individual in maintaining the targeted power output. Maximum stimulation output was limited to the pulse duration corresponding to the participant’s maximum tolerable level. If the participant cycled more than prescribed, then the stimulation ramped down until the prescribed power level was reached. If the participant was able to maintain the prescribed output, then the stimulation ramped down to sensory levels. If the participants could not initially cycle continuously for 30 min when they started the cycling protocol, they were asked to cycle for as long as possible, with brief rest breaks until a total exercise time of 30 min was attained. If a participant missed a training session during the week, make up sessions were allowed as long as the sessions did not exceed 4 times per week.

##### VOL Group

This group used the same cycling setup as the FES group but did not receive any stimulation during training. They also followed the same cycling protocol of 30 min three times a week for 8 weeks while maintaining a 50–80% Karvonen-predicted target heart rate zone.

##### CON Group

This group did not participate in any cycling intervention during the 8-week period.

### 2.5. Outcome Measures

Assessments of cardiorespiratory fitness were conducted for all three groups at three time points: prior to training (PRE), at the end of 8 weeks of training (POST), and during a washout period of 8 weeks after the cessation of the training protocol (WO). An additional assessment was performed midway through training to account for increased cardiorespiratory capacity and motor learning effects, and new HR and power targets were set.

The outcome measures included: (a) cadence (rpm, number of cycling revolutions completed in one minute), (b) peak VO_2_ (liters of oxygen per minute per kg body weight), and (c) peak net HR (peak HR in beats per minute (bpm) during exercise-resting HR). Resting baseline measurements of peak VO_2_ and heart rate were taken for five minutes. Each participant’s maximum power output and mean cadence was determined from an all-out pedaling trial, where the participants pedaled as hard and as fast as they could for one minute, typically performed the day before to avoid fatigue prior to the subsequent incremental load test. For the incremental load test, participants first performed a “warm-up” phase. They sat relaxed in the seat and did not pedal actively while the auxiliary motor turned the crank passively at the mean cadence of the all-out pedaling trial. At the end of the first minute the motor was turned off and the participants began the “exercise phase” of pedaling actively while watching the visual feedback animation on the laptop to maintain the target power output. The power targets started at 10% of their maximum power from the all-out trial and increased by 10% every minute, until the subject was no longer able to maintain the power output target, became too tired to continue, or exceeded a respiratory equivalent ratio greater than 1.0. Following the exercise phase, participants completed a 1 min cool down period of passive cycling with the motor on followed by a 5 min recovery period. During the test, cadence, HR, and breath by breath VO_2_ measurements were recorded and subsequently analyzed. An increase in these measures indicated an improvement in cardiorespiratory fitness. 

### 2.6. Statistical Analysis

Two individuals in the FES group were lost at follow-up due to sickness or inability to come for assessment. Both VO_2_ and HR data were smoothed using a moving average window of 15 s, and peak values were then selected. For computing the peak net rise in HR, resting HR on the day of testing was subtracted from the peak HR. Cadence was computed as the number of complete revolutions during the incremental test divided by time after excluding the warm-up and cool-down revolutions. 

Analyses were conducted using a latent growth curve model (LGCM) in Mplus, (version 8.5, Muthe’n and Muthe’n, Los Angeles, CA, USA) [19]. LGCM is a growth curve analysis that models how individual trajectories of change over time (slope) differ for different groups of individuals to examine changes in an outcome measure over time. Traditional analyses of mean change (e.g., repeated measures ANOVA) examine the difference in the group means (and not the slope or the growth trajectory) at different time points; more importantly, they only use those participants in the analysis who have data across all the time points. This results in loss of information from participants with missing data at one or more time points, a highly likely scenario during a 16-week long study. LGCM, by using a maximum likelihood estimation for handling missing data, uses all of the participants in the analysis. It is thus more robust to partially missing data and unequally spaced time points; hence, it was the method of choice for data analysis. 

Between group differences for FES, VOL, and CON were assessed on the within-person change, i.e., the slope or the growth curves between PRE to POST and POST to WO for cadence, peak VO_2,_ and peak net HR. The loadings of the intercept (starting point) factors in the model were not fixed, allowing each participant to have their own initial level of cadence, peak VO_2,_ and peak net HR. The residual variances were freely estimated for all three outcome measures. Based on the recommendations by Feingold [20], effect sizes were estimated at the POST and WO by dividing the difference between the experimental and control group mean growth rates by the standard deviation at baseline. The resultant effect sizes that were produced for a growth curve model (d_GMA_) were analogous to those generated by the traditional methods using Cohen’s d with the following interpretation criteria: small (0.20), moderate (0.50), and large (0.80).

## 3. Results

**Figure 1:**
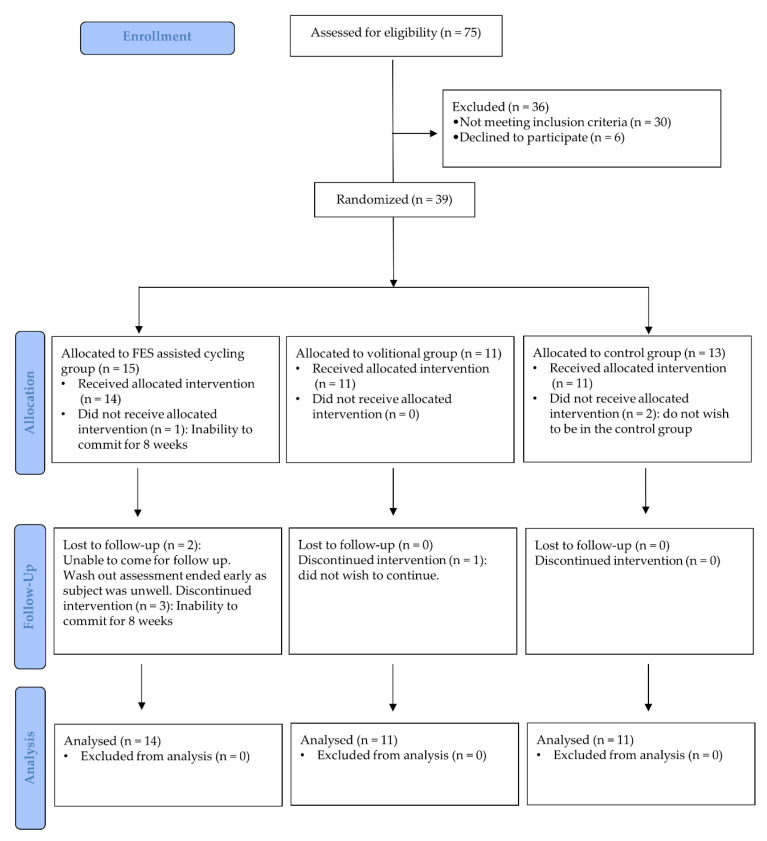
Participant flow diagram illustrating the number of participants who were randomly assigned, received the intended treatment, and analyzed for the cardiovascular outcomes for each group.

Figure 1 summarizes the trial flowchart for this study. Thirty-nine participants enrolled in the study (15 FES, 11 VOL, 13 CON). The patient characteristics at baseline (Table 2) showed no significant differences in age, height, weight, and BMI among the three groups on one-way ANOVA (*p* = 0.300, *p* = 0.389, *p* = 0.081, *p* = 0.133, respectively). The average adherence to the training protocol in both the cycling groups was 91.9%, with no significant difference between the FES and VOL groups (*p* = 0.118). 

**Figure 2:**
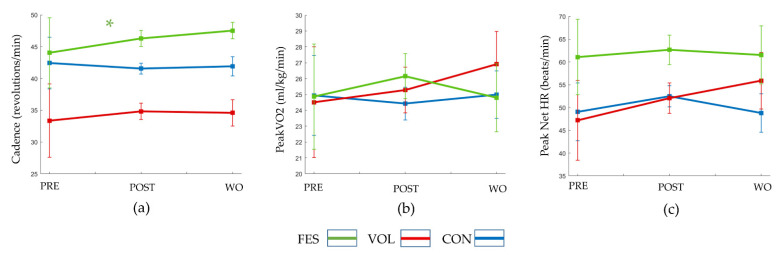
Cardiovascular performance during incremental test in the functional electrical stimulation-assisted cycling (FES), volitional cycling (VOL), and control (CON) groups. The graphs depict the slope of the (**a**) cadence, (**b**) peak VO_2,_ and (**c**) peak net HR across PRE to POST and POST to washout (WO) time points. Error bars depict standard error of the mean at the three time points.

Table 3 depicts the descriptive statistics, including the means and 95% confidence intervals, for cadence, peak VO_2,_ and peak net HR across PRE, POST, and WO. The results of the LGCM that estimated the slope of the outcome measures across PRE to POST and POST to WO time points (Figure 2) are explained for each outcome below:

### 3.1. Cadence

The results of the model show that over 8 weeks of training from PRE to POST, the CON group experienced a decline in cadence (B = −0.865, *p* = 0.402), while the VOL group experienced a small increase (B = 1.455, *p* = 0.204), both of which were not significant. Conversely, the FES group experienced a statistically significant increase at POST (B = 2.238, *p* = 0.041), which was also significantly higher compared to the CON group (B = 3.104, *p* = 0.039). The effect size for the difference between FES and CON group was small (d_GMA_ = 0.4). The increase in the VOL group was not statistically significantly different from the increase observed in the FES group (*p* = 0.622) and the slight decrease observed in the CON group (*p* = 0.132). The effect size for the difference between VOL and CON was small (d_GMA_ = 0.3).

At the end of the washout period, all three groups (CON: B = 0.343, *p* = 0.840; VOL: B = −0.210, *p* = 0.903; FES: B = 1.244, *p* = 0.487) showed minimal changes in the slope of the mean cadence between POST to WO. Therefore, the slope of the cadence line between POST to WO changed very little, i.e., it stayed relatively horizontal. Since VOL and CON did not make significant gains between PRE to POST, a minimal change in the slope between POST to WO indicated that they maintained the same status quo over WO as well. However, a “flat” line between POST to WO for the FES group implied that the gains between PRE to POST were retained in the POST to WO period. There were no statistically significant differences between the change in the VOL and FES group (*p* = 0.559) and in the VOL and CON group (*p* = 0.819). The effect sizes for the difference between FES and CON (d_GMA_ = 0.1) and for the difference between VOL and CON (d_GMA_ < 0.1) were negligible.

### 3.2. Peak VO_2_

Over the 8 weeks of training, the CON group experienced a decline (B = −0.502, *p* = 0.681), while both the VOL (B = 0.764, *p* = 0.528) and FES (B = 1.279, *p* = 0.276) groups showed an increase from PRE to POST, with slope differences that were not statistically significant. Small effect sizes were found for difference between FES and CON (d_GMA_ = 0.4) and between VOL and CON (d_GMA_ = 0.3). Over the 8 weeks of the washout period, the CON (B = 0.558, *p* = 0.753) and VOL (B = 1.630, *p* = 0.339) groups experienced a minimal increase, while the FES group experienced a minimal decrease (B = −1.349, *p* = 0.455), neither of which were statistically significant. The effect size for the difference between FES and CON was small (d_GMA_ = 0.2), and for difference between VOL and CON (d_GMA_ = 0.1), it was negligible.

### 3.3. Peak Net HR

Over the 8 weeks of training from PRE to POST, all three groups (CON: B = 3.415, *p* = 0.217; VOL: B = 4.821, *p* = 0.095, FES: B= 1.662, *p* = 0.535) showed a minimal increase that was not statistically significant. The effect sizes for the difference between FES and CON (d_GMA_ = 0.1) and for the difference between VOL and CON (d_GMA_ = 0.1) were negligible.

Over the 8 weeks of the washout period, the CON group (B = −3.669, *p* = 0.467) and FES groups (B = −1.242, *p* = 0.826) demonstrated a decline, while the VOL group experienced an increase (B = 3.842, *p* = 0.475) from POST to WO that was not significant. The effect size for the difference between FES and CON was negligible (d_GMA_ = 0.1), and that for the difference between VOL and CON was small (d_GMA_ = 0.3).

For all three outcome measures across all time points, covariance was not statistically significant, indicating that the initial level of the cadence, peak VO_2,_ and HR was not related to the rate of change (slope) in these measures.

## 4. Discussion

This RCT investigated the benefits of FES-assisted cycling and volitional cycling over a no-intervention control group on cardiorespiratory fitness in children with CP over an 8-week cycling training protocol. Additionally, this study also investigated the ability to retain the training effects on cardiorespiratory parameters after an 8-week washout period. 

The findings from this study partially confirm the hypothesis and indicate that while FES-assisted cycling can enable children with CP to attain higher cycling cadences than a cycling alone protocol or without any intervention, it did not show any significant improvements in peak VO_2_ and peak net HR. Furthermore, all three groups showed minimal changes between POST to WO. It is important to note that the PRE-to-POST changes need to be taken into account while interpreting the results. Because the CON and VOL groups did not show significant changes between PRE to POST, a minimal change between POST to WO indicates that overall, across 8 weeks of training and 8 weeks after the cessation of training, the CON and VOL groups did not change much. However, because the FES group made significant gains between PRE to POST, a minimal change between POST to WO is desirable and is indicative of the ability to maintain the gains made during training. Hence, the results show that FES assistance helped retain the higher gains in cadence, even after the cessation of the training. Higher cadences are a result of improved muscle coordination and timing. FES training may have facilitated motor activation via the improved timing and intensity of muscle contractions. Thus, FES assistance may lead to improved functional movement patterns and pedaling efficiency. Additionally, for changes in the cadence from PRE to POST, the slope of VOL (i.e., the increase from PRE to POST) was not statistically significant from either the slope of the FES group or the CON group. This is because the increase in the VOL was small, and it fell between the higher increase in the FES group and the no increase in the CON group, i.e., the slope of the VOL lies between the more positive slope of the FES group and the slight negative slope of the CON group. Hence, it was not statistically significant from either group. This implies that cycling training without FES may help with attaining a higher cycling cadence compared to no training at all but is not as effective as adding FES assistance to cycling.

Our effect sizes also support that both FES and VOL showed a small effect for the cadence and peak VO_2_, with that of FES group being slightly higher than the VOL group. Increased cycling cadence should ideally lead to corresponding improvements in peak VO_2_ and peak net HR, which was not true for the cohort. Both the FES and VOL group showed slight improvements in peak VO_2_ that were not significant compared to CON. For peak net HR, both the VOL and CON groups showed a slight increase, but FES remained relatively stable. This trend is reflected in the effect sizes for the VOL and FES groups at POST, which were small and negligible, respectively. A possible explanation might be that the participants in the VOL and FES groups were cycling at the 50–80% Karvonen maximum-predicted target HR during the at-home training phase. While it is standard practice to determine training intensities using a percentage of the Karvonen-predicted HRmax, a broad range such as 50–80% may have led to the participants training only at the lower end of this range, which may not have been enough to attain therapeutic benefits. Hence, using a narrower range such as the 70–80% Karvonen maximum predicted HR, which has been recommended as the threshold for cardiorespiratory training in young adults, may be more effective [21]. 

It is important to note that the current study was underpowered to find a statistically significant difference. A priori power analysis revealed a desired sample size of 60, with 20 participants in each group. However, 36 participants were recruited overall (N = 11 for CON, N = 11 for VOL, N = 14 for FES). Additionally, the cohort comprised of participants with different functional abilities (GMFCS levels II–IV) and high variability at baseline, which was confirmed by the large confidence intervals at baseline. This heterogeneity combined with a relatively small sample size may contribute to a lack of between group differences. High intersubject variability has been a problem in several previous RCTs on children with CP, leading to insignificant between group difference results on some [22,23,24] or all [25] outcome measures. 

This study only stimulated the quadriceps muscle during cycling because it is the main agonist that drives the typical recumbent cycling motion and provides a simple stimulation protocol that the families could execute at home. Johnston et al., however, demonstrated that not only do children with CP use the hamstrings in addition to the quadriceps while cycling, but they also have a higher degree of agonist–antagonist co-contraction at the hip, knee, and ankle muscles [26,27]. Thus, an approach where only the quadriceps muscle is stimulated may not have been enough to change the abnormal motor control strategies that prevent children with CP from cycling more efficiently. An RCT by Armstrong et al. showed improved gross motor strength and self-reported measures of goal performance and satisfaction after undergoing an 8-week FES cycling program that stimulated hamstrings, gluteal, gastrocnemius, and tibialis anterior muscles in addition to quadriceps. Adopting a more comprehensive stimulation strategy that stimulates the hip, knee, and ankle muscles might be more beneficial.

## 5. Conclusions

Numerous studies have examined the effect of FES in populations such as those of patients who have experienced stroke and spinal cord injury. This study is the first to investigate the aerobic responses to FES assistance during the cycling motion in children with CP, which can serve as a safe exercise modality for patients with a wide spectrum of functional and ambulatory abilities as well as an enjoyable physical activity for children. The findings indicate that FES assistance can help children with CP attain higher cycling cadences and can help to retain these gains in cadence after the cessation of training. Similar increases were not obtained in the peak VO_2_ and peak net rise in the heart rates, indicating that a minimum heart rate target of 50% of the Karvonen maximum predicted heart rate may be insufficient to attain cardiorespiratory benefits. Thus, higher training intensities may be necessary to obtain improvements in peak VO_2_ and heart rate. Overall, this study provided some support that FES-assisted cycling may facilitate motor gains such as increased cycling cadence in children with CP and may potentially help them attain better fitness levels.

## Figures and Tables

**Table 1:** Inclusion and exclusion criteria.

Inclusive Criteria	Exclusive Criteria
Age 10–18 years. Gross Motor Function Classification Scale II, III, or IV. Adequate range of motion of the hips, knees, and ankles to allow pedaling. Self-reported visuo-perceptual skills and cognitive/communication skills to follow multiple step commands for attending to exercise and data collection. Ability to communicate pain or discomfort with testing and training procedures.	Lower-extremity orthopedic surgery or traumatic fracture within the past 6 months. Lower-extremity joint pain during cycling. Hip, knee, or ankle joint instability or dislocation. Lower-limb stress fractures in the past year. Symptomatic or current diagnosis of cardiac disease as assessed by the American Heart Association guidelines for cardiac history. Current pulmonary disease or asthma and taking oral steroids or hospitalized for an acute episode in the past 6 months. Severe spasticity in legs (score of 4 on the Modified Ashworth Scale). Severely limited joint range of motion or irreversible muscle contractures that prevented safe positioning on the cycle.

**Table 2:** Baseline characteristics of participants. Average age, height, mass, and BMI differences (mean ± SD) between the three groups: FES cycling group, volitional group, and the control group.

Participant Characteristics	FES Cycling (*n* = 14)	Volitional (*n* = 11)	Control (*n* = 11)
Demographics			
Age, y	14.5 (2.4)	12.7 (2.1)	13.7 (2.9)
Males	13	8	9
Anthropometrics			
Height, m	1.53 (0.14)	1.43 (0.14)	1.51 (0.19)
Mass, kg	56.49 (22.6)	38.08 (12.8)	47.05 (17.4)
BMI, kg/m^2^	23.81 (6.2)	18.63 (3.8)	20.85 (8.0)
GMFCS level			
II	6	2	4
III	3	4	4
IV	5	5	3

BMI, body mass index; GMFCS, gross motor function classification system; FES, functional electrical stimulation.

**Table 3:** Means and 95% confidence intervals at PRE, POST, and WO for cadence, peak VO_2,_ and peak net HR.

	Group	PRE			POST			WO		
		Mean	95% CI		Mean	95% CI		Mean	95% CI	
Cadence (rev/min)	FES	44	33	55	46	44	49	48	47	52
	VOL	33	22	45	35	32	37	35	31	39
	CON	42	34	50	42	40	43	42	39	45
Peak VO_2_ (mL/kg/min)	FES	24.9	18.4	31.4	26.2	23.4	28.9	24.8	20.6	29
	VOL	24.5	17.7	31.4	25.3	22.5	28.1	26.9	22.9	31
	CON	24.9	20	29.9	24.4	22.4	26.4	25	22.1	27.9
Peak Net HR (beats/min)	FES	61	45	77	63	56	69	62	49	74
	VOL	47	30	64	52	45	59	56	44	68
	CON	49	37	61	52	48	57	49	41	57

FES, functional electrical stimulation; VOL, volitional; CON, control; WO, washout; VO_2_, ventilated oxygen; HR, heart rate.

## Data Availability

The data presented in this study are available upon request from the corresponding author.
